# Biosensors in Occupational Safety and Health Management: A Narrative Review

**DOI:** 10.3390/ijerph17072461

**Published:** 2020-04-03

**Authors:** Antonio Baldassarre, Nicola Mucci, Luigi Isaia Lecca, Emanuela Tomasini, Maria Julia Parcias-do-Rosario, Carolina Tauil Pereira, Giulio Arcangeli, Paulo Antonio Barros Oliveira

**Affiliations:** 1 Department of Experimental and Clinical Medicine, University of Florence, 50134 Florence, Italy; 2 Hospital De Clinicas, Serviço de Medicina Ocupacional, Porto Alegre 90035-007, Rio Grande do Sul, Brazil

**Keywords:** occupational medicine, biosensors, ergonomics, health and safety, nanotechnology, environmental medicine, e-health, health biomarker, high fidelity data acquisition, digital epidemiology, high-throughput artificial intelligence, complex adaptive systems

## Abstract

A sensor is a device used to gather information registered by some biological, physical or chemical change, and then convert the information into a measurable signal. The first biosensor prototype was conceived more than a century ago, in 1906, but a properly defined biosensor was only developed later in 1956. Some of them have reached the commercial stage and are routinely used in environmental and agricultural applications, and especially, in clinical laboratory and industrial analysis, mostly because it is an economical, simple and efficient instrument for the in situ detection of the bioavailability of a broad range of environmental pollutants. We propose a narrative review, that found 32 papers and aims to discuss the possible uses of biosensors, focusing on their use in the area of occupational safety and health (OSH).

## 1. Introduction

**Figure 1:**
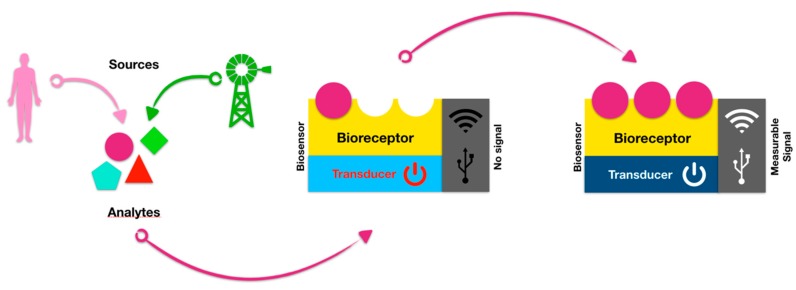
Scheme of a standard biosensor. The biological part is either integrated or closely associated with the physical transducer, and behaves as a recognition element, capable to detect a specific biological analyte, thus generating a measurable signal.

A sensor is a device used to gather information registered by some biological, physical or chemical change, and then convert the information into a measurable signal. Typically, it contains a recognition element and a transducer. Electrochemical biosensors, as a subclass of biological sensors, consist of a biological sensing element and an electrochemical transducer [1]. A biosensor is an analytical device which converts a biological response into an electrical signal, while the term “biosensor” is often used to cover chemical sensor devices used in order to determine the concentration of substances and other parameters of biological interest, even when they do not utilize a biological system directly (Figure 1).

The first chemical sensor that could be considered as a biosensor prototype was conceived about a century ago in 1906, but a properly defined biosensor was only developed in 1956 by Clark et al. Clark subsequently became the father of biosensors because he was able to demonstrate an amperometric enzyme electrode for glucose detection later in 1962. Guilbault and Montalvo implemented a potentiometric biosensor in 1969 by using glass electrodes. Interestingly, Yellow Spring Instruments (YSI) developed the first commercial biosensors in 1975 [2]. Another source of interest in the field of research and development (R&D) is represented by three-dimensional (3D) printing, first patented by Hull in 1986. Since then, numerous 3D printing techniques have been developed. Later on, the concept of including cells into 3D printed constructs emerged, leading to the development of 3D bioprinting [3]. To this end, four-dimensional (4D) bioprinting has been developed using stimuli responsive biomaterials and cell traction forces to make structurally dynamic tissue constructs [3]. Electrochemical biosensors are widely developed, and some of them have reached the commercial stage and are routinely used in environmental and agricultural applications, and especially, in clinical laboratory and industrial analysis [1]. This new technology allows the design of analytical systems, which include a combination of advantages in terms of reduced time-to-result, automation and miniaturization, and consequently portable and user-friendly system platforms for in field high-throughput analysis, detection of a single analyte, as well as simultaneous multi-analyte testing [4]. Electrochemical biosensors can provide large amounts of data in simple, rapid, automated, and relatively inexpensive processes [5,6]. The research, development and use of these and other new techniques, could improve monitoring of environmental pollution and its effects on human health [5]. Toxins in the environment are typically associated with detrimental health outcomes and loss of ecological diversity; therefore, in this scenario, fast and accurate detection of pollutants is pivotal to reduce these threats. Although conventional detection techniques for environmental pollution using physical chemistry methods show a certain degree of sensitivity and specificity, there are still many challenges that limit their practical application, such as expensive equipment and complicated procedures, in addition to long waits for detection [5,7]. Since Sanseverino et al. reported on the design of a whole cell-based biosensor for naphthalene detection in 1990, it has become commonplace for whole cell-based biosensors to be used for bioavailability detection and toxicity assessment of contaminants as these biosensors have been shown to be economical, simple and efficient for the in situ detection of a broad range of environmental pollutants [7]. These biosensors can be classified into three types based on differences in their molecular, cellular and tissue sensing components [7]. The first ones are the molecular-based biosensors, biological active substances such as enzymes, DNA, antigens, antibodies and biofilms as the reporter element. They have high selectivity but expensive macromolecule isolation costs, limited detection capability and short useable lifetime of the identifying molecules. Cellular and tissue sensing biosensors, on the other hand, record information related to the pharmacology, cell physiology and toxicology. Because of the obvious advantages of whole cell-based biosensors such as their good sensitivity, high selectivity and their capability for high-throughput in situ detection, they have been successfully applied to fields such as environmental monitoring, food analysis, pharmacology and drug screening [7]. Nowadays there are different fields of applications for those biosensors, from high-performance liquid chromatography (HPLC) to a whole cell-based biosensor for detection of Pseudomonas putida (BMB-PL), to determination of phenanthrene (PHE) added to red soil samples [7]. Another study, realized by Pasi et al., also tested the bioavailability of lead (Pb) and copper (Cu) concentrations in natural soil using a similar bioluminescent whole cell biosensor, reporting results that were consistent with the results demonstrated by the PHE study; a much higher selectivity was obtained using the whole cell-based biosensor [7]. Recently, a speaker dimension-like biosensor prototype was created for a laptop, controlled by simply plugging in a smartphone. The “two3” device is capable of multiplexed detection of six nucleic acid targets per run. Tests are analyzed onboard in real time, by simply popping open the top, adding sample tubes, pressing start to run the test, and instantly reading the results on the smartphone display [8]. Those devices are also used for analysis of pyrethroid insecticides, the use of which has increased over the last decades, implicating serious concerns about human health. Pyrethroid insecticides are classified as potential environmental endocrine disrupters [9]. Workers such as farmers, pesticide fertilizer spreaders, and manufacturers may be professionally exposed via inhalation and dermal contact [9]. Compared to conventional chemically based analysis which are more expensive, time-consuming and require a specialized laboratory [7], these whole cell biosensors represent a real-time, rapid and unique diagnosis tool, in order to guarantee an improved public health policy, also with a return in occupational health. The new era of the use of biosensors for diagnosis, monitoring and follow-up treatment is already in the general medicine but what are the possible advancements especially for use in occupational medicine? Could these devices provide a new way to help workers’ health?

## 2. Materials and Methods

We propose a narrative review of the literature. The choice for this type of review was carried out in order to provide a broader search of the existing literature and a comprehensive description of a given theme, allowing the identification of gaps in scientific knowledge.

The PICO strategy was performed as follows:

- Problem: Workers monitorization;
- Interest: Use of biosensors;
- Context: Occupational health;
- Outcome: Possible use in Occupational Safety and Health (OSH).

The strategy research, based on PubMed, was defined as follows:

(((((biosensors[Title/Abstract]) OR bioprobes[Title/Abstract]) OR wearable device[Title/Abstract]) OR smartwatch[Title/Abstract])) AND (((((((((((workplace[Title/Abstract]) OR occupational settings[Title/Abstract]) OR occupational medicine[Title/Abstract]) OR occupational health[Title/Abstract]) OR workers[Title/Abstract]) OR job task[Title/Abstract]) OR work[Title/Abstract]) OR worker health[Title/Abstract]) OR work environment[Title/Abstract]) OR environmental exposure[Title/Abstract]) OR exposure assessment[Title/Abstract])

**Figure 2:**
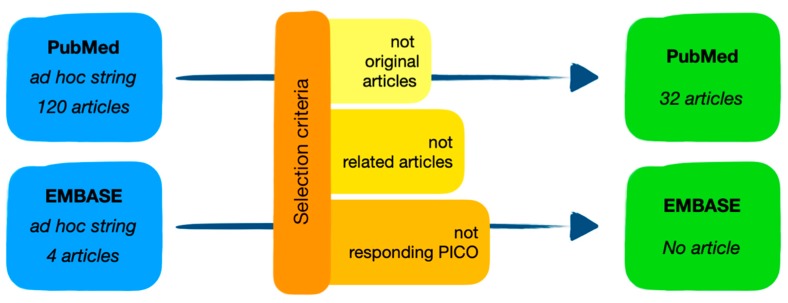
Research strategy flow-chart.

We obtained 120 search results. As inclusion criteria we applied research filters, namely articles with abstract or full production available online and open access in the period between 2010–2019. We excluded not original articles, articles not related to the theme and that did not answer the research question as identified in the PICO criteria. After applying the exclusion criteria, 32 articles were selected from the 120 search results, answering the review question, as reported in Figure 2. The survey was conducted in November 2019.

We also tried to run a research on EMBASE with another ad hoc string:

*(‘‘worker’’ / exp OR ‘‘laborer’’ OR ‘‘laborer’’ OR ‘‘worker’’) AND (‘‘biosensor’’ / exp OR ‘‘biosensor’’ OR ‘‘biosensors’’ OR‘‘ sensor, bio ’’) AND (‘‘ occupational health ’’/ exp OR‘‘ health, occupational ‘‘OR‘‘ occupational fitness’’ OR ‘‘occupational health’’ OR ‘‘professional health’’) AND ‘‘occupational health’’ / exp*

We obtained only 4 search results with no article matching our PICO criteria.

## 3. Results

Just 32 articles, summarized in Table 1, including key messages, met the PICO criteria adopted for this narrative review, as they are eligible for environmental and individual monitoring thanks to the use of biosensors within the occupational health and safety process.

## 4. Discussion

The aim of environmental and personal toxicological monitoring and assessment is to determine the effect of pollution on health. Traditionally, the diagnosis of disease was based on a single diagnostic test. However, modern clinical practice bases the diagnosis of disease on the synthesis of data from several sources [5].

Biosensors, compared to conventional chemically-based sensors, can provide real-time, rapid and unique data [7] and they have a wide range of potential uses for personal toxicity testing and environmental assessments [5], proving to also be useful in the area of occupational medicine, to guarantee workers’ safety and health. They could be used, for example, for the detection of many substances that workers are exposed to, such as organic noxae (e.g., bacterial toxins, mycotoxins and hormones) and inorganic ones (e.g., pesticides, and heavy metals) [5].

It is widely known that personal toxicity testing, mostly used in occupational medicine, requires rapid and accurate detection of a diverse range of chemicals, often present in small traces, and analysis of the interactions of these *noxae* with human health. This remains a formidable challenge [5].

Currently, smartphones are used for a wide range of scientific and medical purposes [8]. Mechanical engineer David Erickson and nutritional scientist Saurabh Mehta, both at Cornell University in Ithaca, New York, have developed a smartphone-based system called the NutriPhone that can detect micronutrients such as vitamin B12 and iron in blood [8]. Not only with smartphones, there are also some studies that developed wearable devices, that can check heart rate, respiration, and accelerations to estimate energy expenditure [18,30], emotion recognition [22] and includean activity tracker [28].

Smart watches are rapidly penetrating the health informatics research space. These studies for health and wellness applications reported encouraging results but were characterized, mainly, by small samples size. Smartwatch measurements, indeed, must be further validated in larger scale studies to understand reliability of collected data [29].

Among the variety of biosensors available today, through physiological and environmental sensors, it is possible to perform detection of real-time asthma attacks [26] as well as the diagnosis of early COPD based on sputum viscosity [19] and, by using electrochemical biosensors, the screening of antipsychotic drugs (APDs) can also be performed [23].

These types of devices, besides the uses already researched, could also be used for other forms of monitoring, to protect the health of workers, helping also in the early diagnosis of work-related diseases and subsequently allow the occupational physician to protect workers’ health against further risks.

The future of these new technologies is bright but there are some facts that still need to be deepened. Most environmental samples tested contain a large number of pollutants as well as other naturally occurring molecules that can mask the signal coming from the analyte of interest. Another issue is the toxic nature of the samples, as these can contain heavy metals or organic pollutants, and their presence can limit the choice of cells used on those microbes resistant to their action. Finally, emission from whole cell-based sensors employed for extended periods of time can become unstable over time as these cells undergo leakage or diffusion [7]. Careful studies should be undertaken to evaluate their use in occupational medicine, as most analysis performed in this area may fit this sample contamination requirement.

But, what can we expect in new advancements of these devices? Despite being a field already well studied, the area related to biosensors is still on its journey and there is much more to be developed besides increasing its applicability into several sectors. Some researchers are studying the use of a portable whole cell biosensor system for environmental monitoring or even the fabrication of specific and multifunctional biosensors for rapid and real-time detection in extreme unfriendly environments [7]. Further studies for the advancement of these technologies should continue to be carried out, especially in the area of occupational safety and health where there is still a lack of new approaches for prevention and diagnosis.

## 5. Conclusions

This narrative review has summarized advancements in the development of biosensors and their possible applications. Machine learning can further increase the accuracy by extracting more features from various biosensors. Future technology-supported interventions for health and wellness will require the data collected from biosensors, which should be integrated with other sources of information. Data, in fact, must be represented as meaningful information for health-related decision-making by a range of stakeholders including patients, family members, health care providers, public health professionals and, last but not least, policy makers.

The application of biosensors in the field of occupational health and safety is far from a reality. The push of industry 4.0 has certainly confirmed a step forward, for example thanks to the extensive use of wearable biometric sensors to monitor workers’ health, towards the application of biosensors.

We firmly believe that technology, if applied in compliance with ethical principles as well, can contribute to improving the health and safety of workers, filling some gaps, such as for work in solitude, with the consequent further reduction of occupational risks.

More research is needed to clarify the biosensors’ use in the occupational medicine area. These studies will have to consider the differences in health disparities, health literacy and technology access, influenced by demographic factors such as socioeconomic status, rural versus urban living situation, gender, age, race and ethnicity.

## Figures and Tables

**Table 1:** Research strategy selected articles.

	Title	Year	1st Author	Method	Key Messages	PICO Criteria
1	Disease-Related Detection with Electrochemical: A Review [1]	2017	Huang Y	Narrative review	Disease-related detection with electrochemical biosensors	P ☐ I ☑ C ☑ O ☐
2	A Review on Carbon Nanotubes in Biosensor Devices and their Applications in Medicine [2]	2018	Sireesha M	Narrative review	Carbon nanotubes (CNTs) biosensors	P ☐ I ☑ C ☑ O ☐
3	Multiplex Biomarker Analysis Biosensor for Detection of Hepatitis B Virus [3]	2015	Xu H	Scientific report	In vitro study protein biosensor for the detection of the hepatitis B virus (HBV)	P ☐ I ☑ C ☑ O ☐
4	Toward the Development of Smart and Low Cost Point-of-Care Biosensors Based on Screen Printed Electrodes [4]	2015	Ahmed MU	Narrative review	Biosensor applications in environmental analysis	P ☐ I ☑ C ☑ O ☐
5	Biosensor and Enviromental Health [5]	2012	Preedy VR Pattel VB	Book (state-of-the-art)	General information - history	P ☐ I ☑ C ☐ O ☑
6	How cutting-edge technologies impact the design of electrochemical (bio)sensors for environmental analysis. A review [6]	2016	Arduini F	Narrative review	Screen printed electrodes	P ☐ I ☑ C ☑ O ☐
7	The Application of Whole Cell-Based Biosensors for Use in Environmental Analysis and in Medical Diagnostics [7]	2017	Gui Q	Narrative review	Whole cell-based biosensors in the areas of pollution detection in environmental and in biomedical diagnostics	P ☑ I ☑ C ☐ O ☑
8	Technology features - Pocket laboratories [8]	2017	Perkel JM	Scientific report	Mobile phone use as laboratory-based science	P ☑ I ☑ C ☐ O ☑
9	Immunoassays and Biosensors for Monitoring Environmental and Human Exposure to Pyrethroid Insecticides [9]	2011	Ahn KC	Narrative review	Immunochemical approaches for the detection of pyrethroid insecticides	P ☐ I ☑ C ☑ O ☐
10	Paper Electrodes for Bioelectrochemistry: Biosensors and Biofuel Cells [10]	2015	Desmet C	Narrative review	Electrochemical paper- based biosensors	P ☐ I ☑ C ☐ O ☑
11	Inkjet Printing for Biosensor Fabrication: Combining Chemistry and Technology for Advanced Manufacturing [11]	2012	Li J	Narrative review	Inkjet biosensor fabrication	P ☐ I ☑ C ☐ O ☑
12	Plug-and-Play Metabolic Transducers Expand the Chemical Detection Space of Cell-Free Biosensors [12]	2019	Voyvodic PL	Narrative review	Cell-free transcription/translation (TXTL) systems	P ☐ I ☑ C ☐ O ☑
13	Detection of Stress Using Biosensors [13]	2018	Singh SA	Clinical trial	A sensor based biological method of stress measurement	P ☑ I ☑ C ☑ O ☐
14	Point of Care Testing: The Impact of Nanotechnology [14]	2016	Syedmoradi L	Narrative review	Point-of-care (POC) diagnostic devices	P ☐ I ☑ C ☐ O ☑
15	Printed Organo-Functionalized Graphene for Biosensing Applications [15]	2016	Wisitsoraat A	Narrative review	Organo-functionalized graphene and printed biosensor	P ☐ I ☑ C ☐ O ☑
16	Advances in Point-of-Care Technologies for Molecular Diagnostics [16]	2017	Zarei M	Narrative review	Miniaturization, nanotechnology, and microfluidics, along with developments in cloud-connected point-of-care (POC) diagnostics technologies	P ☐ I ☑ C ☐ O ☑
17	Advances in Molecularly Imprinting Technology for Bioanalytical Applications [17]	2019	Li R	Narrative review	Molecularly Imprinted Polymers (MIPs) bioprobes and biosensors	P ☐ I ☑ C ☐ O ☑
18	Fusion of Heart Rate, Respiration and Motion Measurements from a Wearable Sensor System to Enhance Energy Expenditure Estimation [18]	2018	Lu K	Experimental clinical trial	A new method that integrates heart rate, respiration, and motion information obtained from a wearable sensor system to estimate energy expenditure	P ☑ I ☑ C ☑ O ☐
19	Design and Fabrication of a BiCMOS Dielectric Sensor for Viscosity Measurements: A Possible Solution for Early Detection of COPD [19]	2018	Zarrin PS	Clinical trial	Complementary metal-oxide semiconductor (CMOS) based dielectric sensor for the real-time monitoring of sputum viscosity as early diagnosis of COPD	P ☐ I ☑ C ☐ O ☑
20	Validation of the AppleWatch for Heart Rate Variability Measurements during Relax and Mental Stress in Healthy Subjects [20]	2018	Hernando D	Clinical trial	Validation of the AppleWatch in terms of patient monitorization	P ☐ I ☑ C ☐ O ☑
21	Thermal Energy Harvesting on the Bodily Surfaces of Arms and Legs through a Wearable Thermo-Electric Generator [21]	2018	Proto A	Experimental clinical trial	Measurements on thermal energy harvesting through a wearable thermo-electric generator (TEG)	P ☑ I ☑ C ☑ O ☐
22	Coverage of Emotion Recognition for Common Wearable Biosensors [22]	2018	Hui T	Experimental clinical trial	Emotion recognition using common off-the-shelf wearable biosensors	P ☐ I ☑ C ☐ O ☑
23	Mining the Potential of Label-Free Biosensors for In Vitro Antipsychotic Drug Screening [23]	2018	Kilik K	Experimental clinical trial	Electrochemical biosensors for the screening of antipsychotic drugs (APDs)	P ☐ I ☑ C ☐ O ☑
24	Re-usable Electrochemical Glucose Sensors Integrated into a Smartphone Platform [24]	2018	Bandodkar AJ	Experimental clinical trial	New smartphone-based reusable glucose meter	P ☐ I ☑ C ☐ O ☑
25	Emerging Strategies and Applications of Layer-by-Layer Self-Assembly [25]	2014	Rawtani D	Narrative review	Layer-by-layer self-assembly	P ☐ I ☑ C ☐ O ☑
26	Feasibility of a Secure Wireless Sensing Smartwatch Application for the Self-Management of Pediatric Asthma [26]	2017	Hosseini A	Experimental clinical trial	Real-time asthma attack through physiological and environmental sensors	P ☐ I ☑ C ☐ O ☑
27	Antibody-Conjugated Gold Nanoparticle-Based Immunosensor for Ultra-Sensitive Detection of Troponin-T [27]	2014	Jacobs M	In vitro study	In-vitro study nanoparticles for detection of troponin	P ☐ I ☑ C ☐ O ☑
28	Evaluating the Effectiveness of Organizational-Level Strategies with or without an Activity Tracker to Reduce Office Workers‘ Sitting Time: A Cluster-Randomized Tria [28]	2016	Brakenridge CL	Cluster-randomized trial	Activity tracker to reduce sitting amongst office workers	P ☑ I ☑ C ☑ O ☐
29	Health at Hand: A Systematic Review of Smart Watch Uses for Health and Wellness [29]	2016	Reeder B	Systematic review	Smart watches	P ☐ I ☑ C ☐ O ☑
30	Estimation of Thermal Sensation Based on Wrist Skin Temperatures [30]	2016	Sim SY	Experimental clinical trial	Thermal sensation estimation technology based on wrist skin temperatures	P ☐ I ☑ C ☐ O ☑
31	Photonics-on-a-Chip: Recent Advances in Integrated Waveguides as Enabling Detection Elements for Real-World, Lab-on-a-Chip Biosensing Applications [31]	2011	Washburn AL	Narrative review	Grating-coupled, interferometric, photonic crystal, and micro resonator waveguide sensors.	P ☐ I ☑ C ☐ O ☑
32	Advances and Future Perspectives in 4D Bioprinting [32]	2018	Ashammaki N	Narrative review	4D bioprinting	P ☐ I ☑ C ☐ O ☑
